# Reference Values of Absolute and Relative Handgrip Strength in Chilean Schoolchildren with Intellectual Disabilities

**DOI:** 10.3390/children9121912

**Published:** 2022-12-07

**Authors:** Claudio Farías-Valenzuela, Paloma Ferrero-Hernández, Gerson Ferrari, Sebastián Espoz-Lazo, Antonio Castillo-Paredes, Sebastián Álvarez-Arangua, Pedro Valdivia-Moral

**Affiliations:** 1 Instituto del Deporte, Universidad de las Américas, Santiago 9170022, Chile; 2 Department of Didactics of Musical, Plastic and Corporal Expression, Faculty of Education, University of Granada, 18071 Granada, Spain; 3 Facultad de Educación y Cultura, Universidad SEK, Santiago 7520318, Chile; 4 Sciences of Physical Activity, Sports and Health School, University of Santiago of Chile (USACH), Santiago 9170022, Chile; 5 Facultad de Ciencias de la Salud, Universidad Autónoma de Chile, Providencia 7500912, Chile; 6 Facultad de Ciencias para el Cuidado de la Salud, Universidad San Sebastian, Lota 2465, Providencia 7510157, Chile; 7 Grupo AFySE, Investigación en Actividad Física y Salud Escolar, Escuela de Pedagogía en Educación Física, Facultad de Educación, Universidad de Las Américas, Santiago 8370040, Chile; 8 Exercise and Rehabilitation Sciences Institute, School of Physical Therapy, Faculty of Reahabilitation Sciences, Universidad Andres Bello, Santiago 7591538, Chile

**Keywords:** handgrip strength, absolute strength, relative strength, reference values, intellectual disability, schoolchildren

## Abstract

Handgrip strength is a simple measure of general muscle strength and is related to functionality in people with intellectual disabilities. The objective of this research was to describe the normative values of absolute and relative handgrip strength in children, adolescents and adults according to sex. The sample was made up of 264 schoolchildren (n = 168 men) belonging to five special education schools in Santiago of Chile. The results show higher levels of absolute handgrip strength in males compared to females. The maximum peak of the absolute manual handgrip is reached in females in adolescence with a decrease in adulthood. Relative handgrip strength levels are similar in boys and girls. In females, the relative handgrip strength is similar in childhood and adolescence. Relative handgrip strength declines in both sexes from adolescence to adulthood. The reference values of this study can be used by professionals in the areas of health and education as a guide for interpretation, monitoring and follow-up of Chilean schooled people with intellectual disabilities.

## 1. Introduction

Muscular strength (MS) is considered a predictive measure of independence and autonomy in activities of daily living [1]. In this sense, people with intellectual disability (ID) have lower levels of voluntary muscle activation [2], a situation that triggers the reduction in MS in a generalized way, associating it with a functional deterioration from the school stage [3].

One of the most widely used measures to quantify MS in a safe, reliable and reproducible way is the handgrip strength (HGS) [4], considered an irreplaceable physical test of muscle function [5]. The HGS in people with ID who attend school is lower when compared to children with typical development [6], according to several existing investigations in the literature of HGS normative values for people without disabilities [7,8,9]. These values present differences when establishing comparisons in HGS due to the lack of standardization of protocols and methodologies used for assessment, as well as the different technologies associated with the diversity of existing dynamometers, making it difficult to extrapolate results from one population to another [10]. In the search for a comprehensive assessment of people with disabilities, various physical condition assessment batteries for people with ID [11,12,13] consider the HGS as a test that measures maximum isometric strength, as well as the dimensions related to the fitness and health of this population.

As a result of the COVID-19 pandemic, schoolchildren with ID reduced absolute HGS by up to 40% in females and 30% in males, adding to the increase in body weight in both sexes compared to a pre-confinement situation [14]. Lower absolute and relative HGS levels are related to increased cardiometabolic risk factors [15], lower survival in cancer patients [16] and risk of mortality from any cause [17]. The reduction in strength levels and body weight rise is an increasingly frequent phenomenon in schoolchildren that occur since childhood and which conjugation compromises relative strength levels, with the consequent increase in the risk of sarcopenic obesity [18]. In addition to the above, Faigenbaum and Meadors [19] declare a phenomenon called “pediatric dynapenia” that corresponds to a decrease in MS from school stages [20] not related to pathological conditions, which affects motor skills, self-confidence and social relationships, leading to a predisposition of people with ID to present cardiometabolic diseases prematurely. On the other hand, people with ID who demonstrate higher levels of HGS also develop better functional capacity, which is reflected in better performance in activities of daily living, such as sitting down and getting up from a chair, moving faster and changing directions [3]. In this sense, female adolescents with ID present lower levels of absolute and relative HGS compared to male adolescents of the same age, a situation that compromises functional capacity, increasing the cardiometabolic risk over the opposite sex [21].

Special education in Chile considers the attendance of children, adolescents and adults between 5 and 25 years of age, who mostly present ID, considering an intelligence quotient (IQ) (≤69) [22]. They are evaluated and classified by specific scales, depending on the chronological age, differentiating their use in children, adolescents [23] and adults [24].

The educational institutions for people with disabilities should prepare them for the acquisition of autonomy and necessary functional independence, which will allow them to develop and, as far as possible, acquire an occupation that commonly requires MS. In this way, it can act as a facilitator of neuromuscular actions when standing [25], associated with carrying out work activities [26]. Prospective studies that analyze HGS behavior over time present different trends in the follow-up of children, adolescents [27] and adults [28]. Despite the information presented, the scientific literature is scarce in relation to absolute and relative HGS normative values in people with ID who attend school, as well as its incidence during the school stage. Therefore, this research aims to describe the normative values of absolute and relative HGS differentiated by sex and age group in Chilean schoolchildren with ID.

## 2. Materials and Methods

### 2.1. Design and Sample

This is a cross-sectional and descriptive study. The sample considered a confidence interval of 95% with a statistical power of 80%, the error limit of 5% and the representativeness of the sample of schoolchildren with ID accounted for 68 subjects [29]. The sample was made up of 264 schoolchildren (168 male); with an average age for both sexes of 15.75 years; in an age range between 5 and 25 years. The participants were selected by convenience, belonging to five special education centers in Santiago of Chile. The tutors of the participants signed informed consent to authorize the student’s participation in the different stages of the project. The research procedures were developed in accordance with the principles of the Declaration of Helsinki [30] and with the approval of the ethics committee of the University of Granada, code 2052/CEIH/2021.

The following inclusion criteria were considered: diagnosis of mild or moderate ID (IQ ≤ 69 and ≥49) assessed through the ‘‘Wechsler Intelligence Scale for Children’’ or WISC III [23], and WAIS IV or “Wechsler Intelligence Scale for Adults-IV” [24], diagnosis provided by the psychologist of each educational center, independent mobility, compatible medical certificate of health, active participation in physical education classes (minimum 90 min, once a week) and attendance at evaluations in the company of a family member, older than 18 years. Exclusion criteria were considered: having severe–profound ID, grip difficulties, use of canes or crutches, dependency to perform motor tasks, amputations and/or multiple physical disabilities and wheelchair dependency.

### 2.2. Procedures

The data of the participants were collected between the months of August and November of 2021, within the framework of the anthropometric, physical and functional evaluation of “Ludoinlusión 19”^®^ project belonging to the Vice-rector’s Office for Community Outreach (VIME, in Spanish) of Universidad de Santiago de Chile.

Between the months of June and July, the directors of the participating educational centers were contacted, with whom a meeting was arranged with the participating students’ relatives. In this instance and in a telematic way, the objectives of the project, protocols of the evaluations and the different phases of intervention of the project were explained to them. The families were asked to accompany them in the evaluations, and they were given instructions on the preconditions and requirements for their application. In addition, they were asked to sign the informed consent for the subsequent application of the planned protocol. A call system was organized together with the committed educational centers that allowed the permitted capacity to be maintained during the health situation due to COVID-19, according to Chile’s deconfinement plan. The evaluations were distributed in AM and PM shifts, initially unifying the evaluations for children (5 to 11 years old), followed by adolescents (12 to 17 years old) and, later, adults (18 to 25 years old). In August, the evaluations were carried out through a circuit made up of 3 stations, each student attended in the company of a family member who acted as facilitator of this process. All students began the evaluation tour at station one, corresponding to “Anamnesis and personal history”. Successively, they went to station two, “Anthropometry”, where they measured: body weight (kg), height (cm), waist circumference (cm) and two indexes obtained from the aforementioned evaluations, body mass index (BMI) (kg/m^2^) and waist-to-height ratio (WHtR). Finally, and once this station was completed, they went to station three, for the “Muscular Strength” assessment, where the maximum isometric strength of both upper limbs was measured. The previously recorded data were grouped by sex and according to the ages defined for each age group.

### 2.3. Variables and Instruments

#### 2.3.1. Anthropometry

The body weight of the schoolchildren sample was recorded in kilograms (kg) and height in centimeters (cm). Both measurements were made using a digital scale integrated with a SECA brand stadiometer, model 206. Waist circumference was measured in (cm) with an inextensible metal tape measure (CESCORF^®^, Porto Alegre, Rio Grande do Sul, Brazil) calibrated in centimeters with millimeter graduations. For its evaluation, the midpoint of the distance between the lower costal margin and the upper margin of the iliac crest was considered [31]. From the above anthropometric measurements, BMI (body weight ((kg)/(m^2^)) and WHtR (waist circumference (cm)/height (cm)) were calculated as anthropometric indexes of cardiometabolic risk in people with ID [32].

#### 2.3.2. Muscle Strength

Maximal isometric strength was measured with a hydraulic handgrip dynamometer (Baseline^®^ modelo LiTE^®^, Fabrication Enterprises, Inc., New York, NY, USA) validated with Jamar (J.A. Preston Corporation, Clifton, NJ, USA) [33] using the guidelines established by the American College of Sports Medicine [34]. The participant was tested in a standing position placing the handgrip dynamometer parallel to the side of the body at about waist level. The forearm should be level with the thigh. The subject may flex the arm slightly. The procedure was carried out by two evaluators, one of them delivered the test instructions, indicating that it should be pressed as hard as possible, while the other demonstrated its use. The second joint of the fingers should ‘fit’ under the handle of the handgrip dynamometer. The protocol applied to the students consisted of making three attempts [35], one of familiarization with each limb led by the evaluator, who instructed the student about the correct use and execution. Successively, the student made two attempts of each upper extremity for five seconds, alternately and with a one-minute pause between measurements. Finally, the average of both attempts for each limb was considered the final HGS value for each limb [13]. Subsequently, once the absolute HGS was recorded, the relative HGS (HGS (kg)/body weight (kg)) was calculated [36,37].

### 2.4. Statistical Analysis

The normality of the variables was contrasted with the Kolmogorov–Smirnov test for the entire sample. In the subgroups composed of samples > 50, the previous test was used, while for those less than <50, Shapiro–Wilk test was applied. The total sample was divided into 3 groups (children: 5 to 11 years old; adolescents 12 to 17 years old; and adults 18 to 25 years old). Age, body weight, waist circumference, BMI and WHtR are presented as mean and standard deviation. To establish comparisons of these measurements between the defined groups, the one-way ANOVA test was used for the parametric distribution variables (height and waist circumference) and the Kruskal–Wallis test for the non-parametric distribution variables (age, weight, BMI and WHtR). The Friedman test was used to establish intra- and intergroup comparisons of the absolute and relative HGS between children, adolescent and adult categories and their sex. Finally, the absolute and relative HGS is presented in percentile tables (p5, p10, p25, p50, p75, p90 and p95) according to sex and age group. The statistical program used was SPSS V26 software (SPSS Inc., IBM Corp., Armonk, New York, NY, USA). The significance level adopted was 5%.

## 3. Results

Table 1 presents the anthropometric characteristics of schooled children, adolescents and adults according to sex (mean and standard deviation) of the 264 participants, where 63.6% correspond to males. Of these, 32.1% correspond to boys, 38% to adolescents and 29.9% to adults. Of the female group (36.4%), 31.2% correspond to girls, 36.4% to adolescents and 32.3% to adults. The male students presented age averages (15.34 ± 5.73 years); body weight (57.20 ± 19.84 kg); height (1.55 ± 0.15 m); waist circumference (77.09 ± 18.54 cm); BMI (22.10 ± 7.21 kg/m^2^); and WHtR (0.48 ± 0.12). The female students (n = 96) presented average ages (15.81 ± 5.28 years); body weight (57.29 ± 22.16 kg); height (1.47 ± 0.12 m); waist circumference (80.28 ± 20.24 cm); BMI (23.36 ± 10.89 kg/m^2^); and WHtR (0.50 ± 0.18). Significant differences (*p* < 0.05) are noticed when establishing comparisons between the groups of children, adolescents and adults of both sexes, in the variables: age, body weight, height and BMI. The waist circumference only presents significant differences in females, while the WHtR only presents differences in males.

**Figure 1:**
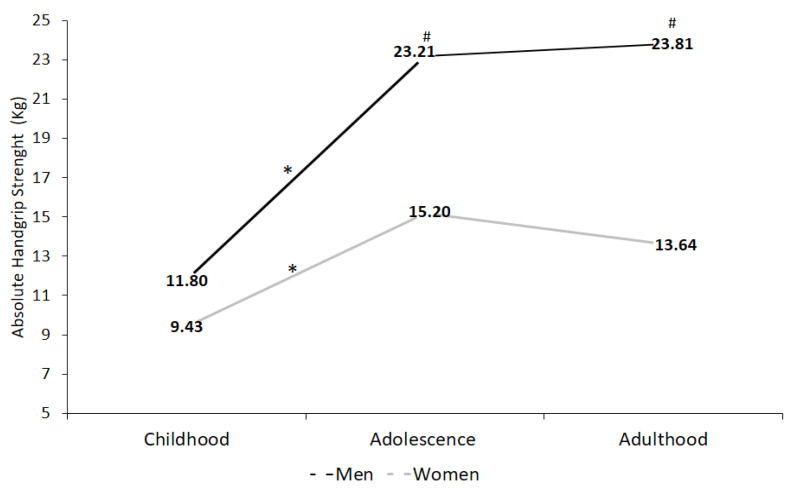
Absolute handgrip strength (HGS) according to sex in the different stages of life course for children, adolescents and adults with intellectual disabilities enrolled in school. Data are presented as medians, significance value *p* < 0.05 used for the Friedman test. * = Intergroup differences; # = Intragroup differences.

Figure 1 presents the behavior of the average absolute HGS of both extremities in children, adolescents and adults, according to sex. The significant increase in absolute HGS from childhood to adolescence is observed in both sexes (male (Δ) % ↑ 49.1; *p* < 0.001 and female (Δ) % ↑ 37.9; *p* < 0.001). However, from adolescence to adulthood there are no significant changes. Females show a reduction in the HGS ((Δ) % ↓ 10.2; *p* = 0.93) from adolescence to adulthood, while males show a tendency to stabilize compared to adolescence ((Δ) % ↑ 2.4; *p* = 0.43). When establishing comparisons of the absolute HGS between sexes, males showed higher levels than females with significant differences in adolescence and adulthood (childhood ((Δ) % ↑ 20.1; *p* = 0.08); adolescence ((Δ) % ↑ 34.5; *p* < 0.001) and adulthood ((Δ) % ↑ 42.6; *p* < 0.001).

**Figure 2:**
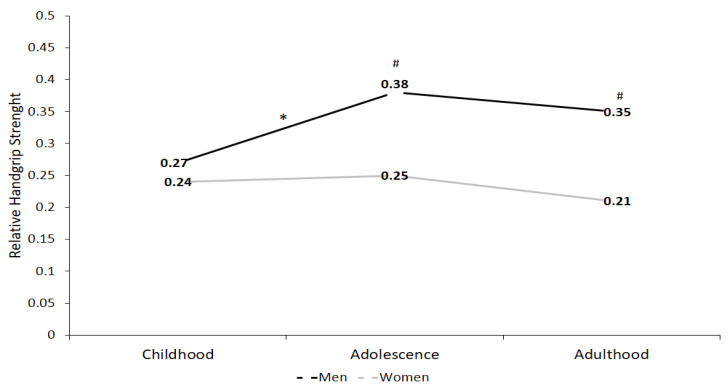
Relative handgrip strength (HGS) according to sex in the different stages of life course for children, adolescents and adults with ID attending school. Data are presented as medians, significance value *p* < 0.05 used for the Friedman test. * = Intergroup differences; # = Intragroup differences.

Figure 2 presents the behavior of the average relative HGS of both extremities in children, adolescents and adults, according to sex. The increase in the relative HGS is observed in males ((Δ) % ↑ 28.9; *p* < 0.001) while in females, a slight increase is observed ((Δ) % ↑ 4.0; *p* = 0.19) in the transition from childhood to adolescence. From adolescence to adulthood, both sexes show a reduction in relative strength levels, (Δ) % ↓ 7.8; *p* = 0.87) in males and a ((Δ) % ↓ 16.0; *p* = 0.43) in females. When establishing comparisons in the relative HGS between sexes, men presented higher levels than females with significant differences in adolescence and adulthood (childhood ((Δ) % ↑ 11.11; *p* = 0.34); adolescence ((Δ) % ↑ 34.21; *p* < 0.001); and adulthood ((Δ) % ↑ 40.00; *p* < 0.001).

Table 2 presents the percentiles (p5, p10, p25, p50, p75, p90 and p95) of absolute and relative HGS of the right and left upper limb in male schoolchildren with ID according to age group. In relation to P50, similar values of absolute and relative HGS are observed from adolescence to adulthood in males in both limbs.

Table 3 presents the percentiles (p5, p10, p25, p50, p75, p90 and p95) of absolute and relative HGS of the right and left upper extremity in female schoolchildren with ID according to age group. In relation to P50, a tendency to decrease in absolute and relative HGS is observed from adolescence to adulthood in females in both limbs.

## 4. Discussion

This study is the first to present reference values of absolute and relative HGS in Chilean schoolchildren with ID between 5 and 25 years of age according to sex. The results show higher levels of absolute and relative HGS in males over females in childhood, adolescence and adulthood. The maximum peak of absolute HGS in females is obtained in adolescence and then declines in adulthood, while in men the absolute HGS in both stages is similar. The relative HGS is also higher in males than females in all stages, with no differences between sex in childhood. The relative HGS behavior in females is similar from childhood to adulthood, while in men there is a significant increase from childhood to adolescence, without significant changes from this stage to adulthood.

The results of this study show lower levels of absolute and relative HGS in people with ID of both sexes during the entire school stage when compared with students without disabilities. The study by Ramirez-Velez et al. [38] in Colombian schoolchildren between 9 and 12 years old recorded absolute HGS values of 14.6 kg and 14.10 kg in boys and girls, respectively, higher than the results of the present investigation, whose values were 11.80 kg in boys and 9.43 kg in girls with ID. Regarding adolescence, this same study revealed average values of 26.23 kg in males and 21.43 kg in females, while in the same age group in Chilean people with ID, the values were 23.21 kg and 15.20 kg, respectively. In agreement with the previous study, the results of Garcia Hermoso et al. [39] in Chilean schoolchildren between 8–12 years old also established higher levels of absolute HGS in boys (16.25 kg) than girls (14.90 kg). Although this agrees with the findings of the present study, in both cases they are greater than the absolute HGS of schoolchildren with ID. When establishing comparisons of the absolute HGS results of Chilean adolescents and adults with ID with other age groups, these present lower values of absolute HGS than those of >85 years older people in a state of dependency [40]. The differences presented could be due to a multifactorial problem in people with ID, which can be approached from an individual or collective perspective, attributing this gap to the predominance of sedentary behaviors and non-compliance with physical activity recommendations [41], the social barriers that make it difficult to access exercise programs and that affect physical condition [42], the use of medications for the treatment of associated comorbidities [43] and intrinsic factors such as a lower neuromuscular capacity for the development of muscle strength [2]. Due to the low development of MS established as one of the fundamental pillars of physical condition and functional capacity together with a cardiorespiratory capacity [44], which also contributes positively as an independent factor to existing comorbidities and the characteristics of people with ID in life expectancy [45], these elements should be a priority in their approach from the school stage.

On the other hand, relative HGS is also lower in people with ID when compared to schoolchildren without ID. Ramirez-Velez et al. [38] present average values of relative HGS of 0.42 in boys and girls and 0.50 in adolescents with an ascending behavior from childhood to adolescence. In a differentiated way, girls present average relative HGS values of 0.39 and 0.42 in adolescence, lower than males. In addition to the above, Garcia Hermoso et al. [39], in a sample of Chilean schoolchildren without disabilities between 8–12 years old, declared the average values of relative HGS of 0.38 in boys and 0.34 in girls. Although there are differences with the previous study carried out in Colombian schoolchildren, it is established that the relative HGS is higher in both studies compared to the relative HGS results of Chilean schoolchildren with ID. On the contrary, the maximum peak of strength in people with ID is found in adolescence, with values of 0.38 in males and 0.25 in females, much lower than those presented by schoolchildren without ID of the same age, a situation that could represent premature aging from school age, with likely reductions in muscle mass and strength in people with ID [46]. In this sense, and considering absolute and relative HGS as markers of cardiometabolic risk, lower levels of absolute and relative HGS in people with ID could increase the risk of cardiometabolic diseases, adding to the existing comorbidities in this population, increasing their prevalence from the school stage [47].

When comparing the obtained results with similar studies in other nations on people with ID, Chilean schoolchildren have lower levels of absolute HGS. The study by Cabeza and Castro [48] described absolute HGS normative values in a sample of Spanish adults with ID between 20 and 59 years old, where males between 20 and 24 years old obtained average absolute HGS values of 31.0 kg, while females reached average values of 21.2 kg. These results are greater than those obtained in the present study, whose declared values correspond to values close to P75 and P90 in males and females, respectively, in Chilean people with ID of the same age. Likewise, the study by Cuesta-Vargas and Hilgenkamp [49] in males and females between 20 and 24 years old belonging to Special Olympics with ID, presented absolute HGS normative values of 18.79 kg in men on the right arm and 19.11 kg on the left arm. In females, absolute HGS values were 28.3 kg on the right arm and 28.89 kg on the left arm. In both sexes, the presented values are higher than those obtained in the present investigation, whose differences could be explained by regular participation in some sports discipline or recreational activities.

Despite the differences exposed in HGS when comparing the results of this study with others of similar characteristics in people with ID, these could be determined by the instruments and evaluation protocols used. (a) The type of dynamometer. Studies in DI report the use of different dynamometers, among which the following stand out: hydraulic dynamometer (Jamar, Bolingbrook, IL, USA) [35,49]; (Takei, Tokyo, Japan) [48,50,51]; (MP 150, California, USA) [52]; (Baseline LiTE, New York, NY, USA) [14]. Other investigations do not declare the type of dynamometer used [53,54,55]. (b) The adjustment of the dynamometer grip. Most of the consulted studies do not consider it [50,51,52,53,54,55], while the study by Cuesta-Vargas and Hilgenkamp [49] used the second position of the dynamometer and Cabeza and Castro [48] performed adjustments based on the size of the subject’s hand. (c) Position of the evaluated. Investigations with the subject in a sitting position [49,51,52,55], others in a standing position [48,50,54] and one non-specific [53] are reported. (d) The number of attempts. Most of the studies analyzed reported the use of three attempts [49,50,52,53,54,55], another uses two [35] and one does not state how many attempts were made [51]. Karatrantou et al. [35] point out that making three attempts reports greater reliability than making one or two for the evaluation of HGS in ID. (e) Pause between attempts. Among the protocols analyzed, defining a one-minute pause between attempts is described by two studies [49,50]; however, several applied protocols do not declare control of this component [48,51,52,53,54,55], which may affect the ability to generate tension in the next attempt. Added to the previously stated elements there are intrinsic factors such as low attention span and instructions follow-up of those evaluated [56], low development of social skills and adaptive behaviors [57] as well as the different associated syndromes and related IQ to DI, reasons that make it difficult to apply extensive and rigorous protocols for the evaluation of HGS.

Different training interventions have shown improvements in HGS in people with ID, whose period and modality of intervention are variable. Calders et al. (citation) [58] implemented a combined strength and resistance training program for 20 weeks, reporting gains in HGS of 9 kg in the intervention group compared to the control group. In addition to the above, the study by Rosety et al. [59] applied a resistance training program structured in a circuit of six exercise stations, with a frequency of 3 days/week for 12 weeks, reporting improvements in HGS. Other shorter interventions applied for only 12 sessions using alternative sports such as Kin-ball, also reported significant increases in HGS [60]. Other strategies that could bring benefits in HGS are those reported by Abe et al. [61], 2022, who point out that interventions involving the family nucleus and the educational environment are strategies to increase physical activity in schoolchildren and consequently HGS.

Among the limitations of the study, it can be pointed out that the selected sample was by convenience, and it is a cross-sectional study that does not allow a follow-up to be established regarding HGS behavior over a period of time. Syndromes related to ID present in some schoolchildren, medication use and complementary information related to the lifestyle adopted by the students and their families during the period of confinement were not considered, the low sample of female schoolchildren by age categories for the presentation of percentiles and the variety of dynamometers and protocols used that must be standardized, should be considered in future research.

Among the strengths of this research, the representativeness of the sample that considers both sexes stands out, with results differentiated by age groups, useful for professionals who work in the educational context and in areas related to sport and health for schooled children with ID. There are no records of similar studies in Chile, which generates an opening for a new and emerging area in topics of inclusion and gender equity in research through prospective studies of the physical condition related to health and functional capacity during the school period.

## 5. Conclusions

Absolute and relative HGS present a different behavior in male and female schoolchildren with ID. In males, absolute HGS increases from childhood to adulthood, while in females, it declines from adolescence to adulthood. In both sexes, the maximum peak of relative HGS is obtained in adolescence. Absolute and relative HGS in people with ID is lower than in schoolchildren without ID, increasing the potential risk of chronic and metabolic diseases prematurely. It is essential to develop initiatives that are developed in the educational context and that are related to MS training, through innovative and adherent methodologies that contribute to the improvement of muscle function in Chilean schoolchildren with ID.

## Figures and Tables

**Table 1:** Anthropometric characteristics of schoolchildren with intellectual disabilities.

	Men				Women			
	(n = 168)				(n = 96)			
Variables	Children	Adolescents	Adults	*p* Value	Children	Adolescents	Adults	*p* Value
	(n = 54)	(n = 64)	(n = 50)		(n = 30)	(n = 35)	(n = 31)	
Age (years)	9.65 ± 2.18	14.52 ± 1.52	22.67 ± 3.61	<0.001 ^a^**	9.86 ± 2.02	15.31 ± 1.67	22.12 ± 2.49	<0.001 ^a^**
Body weigth (kg)	42.93 ± 16.29	60.65 ± 15.34	68.17 ± 19.69	<0.001 ^a^**	41.34 ± 17.46	62.22 ± 23.39	66.8 ± 16.34	<0.001 ^a^**
Height (m)	1.40 ± 0.15	1.62 ± 0.10	1.63 ± 0.09	<0.001 ^b^**	1.37 ± 0.12	1.50 ± 0.10	1.53 ± 0.09	<0.001 ^b^**
Waist circumference (cm)	71.01 ± 13.81	74.98 ± 22.02	86.50 ± 13.89	0.05 ^b^	69.26 ± 14.22	82.50 ± 24.13	88.15 ± 15.54	<0.001 ^b^**
BMI (kg/m^2^)	19.95 ± 6.50	22.50 ± 5.34	23.86 ± 9.29	<0.001 ^a^**	20.20 ± 7.00	25.80 ± 11.46	23.54 ± 12.79	0.04 ^a^*
WHtR	0.49 ± 0.10	0.48 ± 0.07	0.53 ± 0.09	0.03 ^a^*	0.48 ± 0.13	0.57 ± 0.15	0.57 ± 0.24	0.18 ^a^

a = ANOVA one way; b = Kruskal–Wallis. Significance value * *p* < 0.05; ** *p* < 0.001.

**Table 2:** Absolute and relative HGS percentiles in male schoolchildren with ID.

Absolute Handgrip Strength (Kg)								
Rigth Arm								
Age Group	*n*	P5	P10	P25	P50	P75	P90	P95
Children	54	2.35	3.75	6.81	10.50	17.66	25.30	27.95
Adolescents	64	9.00	10.75	16.00	22.65	26.00	39.50	47.87
Adults	50	5.37	9.00	16.75	23.50	32.75	40.35	43.08
Left arm								
		**P5**	**P10**	**P25**	**P50**	**P75**	**P90**	**P95**
Children	54	2.00	3.25	5.00	10.00	17.00	22.25	23.62
Adolescents	64	10.50	11.75	14.87	21.55	25.00	34.75	47.37
Adults	50	2.92	9.86	15.75	21.50	31.75	41.75	44.27
Relative Handgrip Strength								
Rigth arm								
Age group	***n***	**P5**	**P10**	**P25**	**P50**	**P75**	**P90**	**P95**
Children	54	0.01	0.07	0.16	0.25	0.39	0.51	0.62
Adolescents	64	0.16	0.21	0.23	0.37	0.52	0.63	0.78
Adults	50	0.14	0.15	0.24	0.35	0.46	0.63	0.74
Left arm								
		**P5**	**P10**	**P25**	**P50**	**P75**	**P90**	**P95**
Children	54	0.03	0.09	0.13	0.22	0.33	0.50	0.57
Adolescents	64	0.18	0.19	0.24	0.35	0.47	0.54	0.74
Adults	50	0.04	0.16	0.24	0.31	0.46	0.53	0.70

**Table 3:** Absolute and relative HGS percentiles in female schoolchildren with ID.

Absolute Handgrip Strength (Kg)								
Rigth Arm								
Age Group	*n*	P5	P10	P25	P50	P75	P90	P95
Children	30	2.00	2.45	4.00	6.55	12.00	23.68	29.00
Adolescents	35	3.22	3.95	9.12	15.25	20.31	30.10	31.55
Adults	31	4.50	6.25	8.62	13.65	19.37	21.25	23.55
Left arm								
		**P5**	**P10**	**P25**	**P50**	**P75**	**P90**	**P95**
Children	30	0.45	2.41	4.00	7.00	12.55	23.45	29.00
Adolescents	35	3.45	4.00	10.25	14.55	21.45	28.35	31.55
Adults	31	4.31	4.75	9.62	13.55	18.00	20.55	22.55
Relative Handgrip Strength								
Rigth arm								
Age group	***n***	**P5**	**P10**	**P25**	**P50**	**P75**	**P90**	**P95**
Children	30	0.04	0.08	0.11	0.20	0.31	0.53	0.57
Adolescents	35	0.02	0.07	0.18	0.25	0.36	0.42	0.51
Adults	31	0.05	0.10	0.15	0.22	0.28	0.40	0.44
Left arm								
		**P5**	**P10**	**P25**	**P50**	**P75**	**P90**	**P95**
Children	30	0.00	0.03	0.12	0.26	0.33	0.56	0.57
Adolescents	35	0.03	0.07	0.15	0.25	0.36	0.44	0.48
Adults	31	0.06	0.07	0.11	0.22	0.29	0.36	0.39

## Data Availability

The data that support the findings of this study are available from the corresponding author upon reasonable request.
